# Automated versus Chemically Intuitive Deconvolution of Density Functional Theory (DFT)-Based Gas-Phase Errors in Nitrogen Compounds

**DOI:** 10.1021/acs.iecr.2c02111

**Published:** 2022-09-02

**Authors:** Ricardo Urrego-Ortiz, Santiago Builes, Federico Calle-Vallejo

**Affiliations:** † Escuela de Ciencias Aplicadas e Ingeniería, Universidad EAFIT, Carrera 49 # 7 sur 50, 050022, Medellín, Colombia; ‡ Department of Materials Science and Chemical Physics & Institute of Theoretical and Computational Chemistry, University of Barcelona, Martí i Franquès 1, 08028 Barcelona, Spain; § Nano-Bio Spectroscopy Group and European Theoretical Spectroscopy Facility (ETSF), Department of Polymers and Advanced Materials: Physics, Chemistry and Technology, University of the Basque Country UPV/EHU, Avenida Tolosa 72, 20018 San Sebastián, Spain; ∥ IKERBASQUE, Basque Foundation for Science, Plaza de Euskadi 5, 48009 Bilbao, Spain

## Abstract

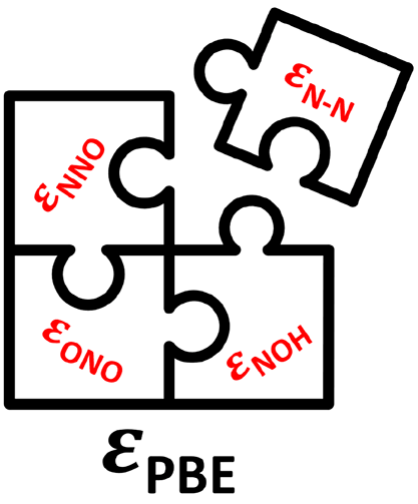

Catalysis models involving metal surfaces and gases are regularly based on density functional theory (DFT) calculations at the generalized gradient approximation (GGA). Such models may have large errors in view of the poor DFT-GGA description of gas-phase molecules with multiple bonds. Here, we analyze three correction schemes for the PBE-calculated Gibbs energies of formation of 13 nitrogen compounds. The first scheme is sequential and based on chemical intuition, the second one is an automated optimization based on chemical bonds, and the third one is an automated optimization that capitalizes on the errors found by the first scheme. The mean and maximum absolute errors are brought down close to chemical accuracy by the third approach by correcting the inaccuracies in the NNO and ONO backbones and those in N–O and N–N bonds. This work shows that chemical intuition and automated optimization can be combined to swiftly enhance the predictiveness of DFT-GGA calculations of gases.

## Introduction

Because of its wide range of oxidation states from −3 to +5, nitrogen forms a wide and diverse group of compounds when combined with hydrogen and oxygen, including oxides, hydrides, radicals, ions, and acids.^1^ All those compounds are part of the nitrogen cycle and are relevant in aquatic and terrestrial systems, atmospheric chemistry, and chemical industries.^2−6^ The reactions connecting these compounds have gained interest in the scientific community, because of their industrial uses, the adverse effect some of them have on human health, their role in climate change, and the colossal imbalance of the nitrogen cycle as a result of human activities.^1,7−11^

The experimental assessment of the chemical properties of nitrogen-containing species is far from straightforward, given their instability/reactivity and the complex reaction networks they form.^12−14^ Hence, density functional theory (DFT) calculations have been widely used to predict structural and thermochemical properties of this family of compounds.^15−18^ To achieve fair predictions of molecular systems, DFT calculations frequently rely on hybrid exchange-correlation functionals, such as B3LYP,^19^ which are computationally expensive and not advisible for systems with delocalized electrons.^20,21^ This is certainly restrictive for studies in areas such as heterogeneous electrocatalysis, where the systems often involve gases and liquids in contact with conductive solids.^22^

Exchange-correlation functionals based on the generalized gradient approximation (GGA) are widely used to study catalytic systems in view of their affordable computational requirements and reasonable predictions of metal bulk and surface properties.^23−26^ Although the limitations of GGAs in describing the energetics of molecules are well-known,^27−29^ semiempirical and fully computational schemes can be devised to rapidly correct them.^30−34^ Correction schemes generally seek to identify specific groups of atoms or functional groups that systematically contribute to the total errors of different molecules. While the errors can mostly be attributed to poor descriptions of the exchange contribution to the total DFT energy in molecules with multiple bonds and/or strongly interacting lone pairs,^27,35^ the detection and quantification of systematic errors enables otherwise modest GGA functionals to produce accurate yet inexpensive predictions.

In this paper, we analyze three approaches to determine the error contributions in the formation energies of 13 compounds containing N, O and H, using the GGA-PBE exchange-correlation functional.^36^ The first approach is sequential and based on chemical intuition, the second approach is automated and based on chemical bonds, and the third approach is also automated and capitalizes on the findings of the first one. The sequential approach finds the errors in a stepwise fashion, while the automated approaches minimize error functions with free parameters. Apart from swiftly bringing the PBE-calculated Gibbs energies close to the experimental values, our results show how chemical intuition can be used as an initial step to identify errors that automated schemes can further minimize.

## Computational Methods

Ball-and-stick representations of the nitrogen-containing compounds studied here (NH_2_OH, NO, HNO, NO_2_, NO_3_, *trans*-HNO_2_, *cis*-HNO_2_, HNO_3_, N_2_O, *cis*-N_2_O_2_, N_2_O_3_, N_2_O_4_, N_2_O_5_) are shown in Figure S1 in the Supporting Information. The DFT calculations were performed using the Vienna ab initio simulation package (VASP),^37^ the Perdew–Burke–Ernzerhof (PBE) exchange-correlation functional,^36^ and the projector augmented-wave (PAW) method.^38^ The plane-wave cutoff for all the calculations was 450 eV, shown previously^30,39,40^ and verified in Figure S2 in the Supporting Information for N_2_O formation to provide converged reaction energies. Gaussian smearing with *k*_B_*T* = 10^–3^ eV was used and all energies were extrapolated to 0 K. During the structural optimization of the molecules, carried out using the conjugate gradient algorithm, all atoms were allowed to relax in all directions until the maximal atomic forces were equal to or smaller than 0.01 eV/Å. The molecules were simulated in large boxes in which the distance between periodic images was at least 12 Å. Accordingly, we only considered the Γ-point for the *k*-point sampling of the calculations. Spin-unrestricted calculations were performed for O_2_, NO, NO_2_, NO_3_, and *cis*-N_2_O_2_. Note that NO, NO_2_, and NO_3_ are neutral free radicals, not anions or cations. The error optimizations were formulated in GAMS using the ANTIGONE^41^ global optimization solver on the NEOS Server.^42,43^

The formation of a generic nitrogen compound H_*x*_N_*y*_O_*z*_ from its elements in their respective standard states is defined as
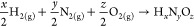
1

If the molecule does not contain hydrogen (e.g., NO_3_), *x* = 0 in eq 1. Likewise, if the molecule does not contain oxygen (e.g., NH_3_), *z* = 0 in eq 1. Following previous works,^30,32,39,40^ the total error in the DFT description of a generic nitrogen compound (ε_H_*x*_N_*y*_O_*z*__^T^) is defined as the difference between the DFT-calculated and the experimental energies of formation, see eq 2. In this case, we will make the analysis in terms of Gibbs energies of formation (Δ_f_*G*_H_*x*_N_*y*_O_*z*__^DFT^ and Δ_f_*G*_H_*x*_N_*y*_O_*z*__^exp^). However, we note that the analysis can be made in terms of enthalpies of formation and the results would be identical, because the total entropies of the molecules are usually taken from tabulated experimental data.^44,45^
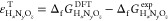
2

The Gibbs energies of formation were approximated by means of DFT as follows:
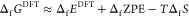
3where Δ_f_*E*^DFT^ is the formation energy calculated with DFT total energies, Δ_f_ZPE the zero-point energy change calculated using DFT within the harmonic oscillator approximation, and *T*Δ_f_*S* the entropy change at *T* = 298.15 K, taken from thermodynamic tables.^44,45^ We did not incorporate heat capacity contributions to the formation energies in eq 3, because their energy change has been shown to be small in the range of 0 to 298.15 K^31,46^ (see further details in section S6 in the Supporting Information). We note that previous works showed that the differences between experimental and calculated Δ_f_ZPE are negligible for various H_*x*_N_*y*_O_*z*_ compounds,^34^ such that the errors can be entirely assigned to Δ_f_*E*^DFT^. The experimental Gibbs energies used in eq 2 to compute the errors are also taken from thermodynamic tables.^44,45^

As shown in eq 4, the total error in eq 2 for H_*x*_N_*y*_O_*z*_ is the difference in the errors of the products and reactants as given by eq 1:
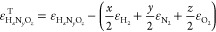
4

In eq 4, ε_H_2__, ε_N_2__, and ε_O_2__ are the respective individual errors of H_2_, N_2_, and O_2_, and ε_H_*__*x*__*_N_*__*y*__*_O_*__*z*__* is the gas-phase error of the generic nitrogen compound. A usual simplification is ε_H_2__ ≈ 0, because H_2_ is generally well described by DFT.^27^ In contrast, ε_N_2__ and ε_O_2__ are generally large^27,47^ and are respectively assessed based on the reactions in which N_2_ is combined with H_2_ to produce NH_3_,^32^
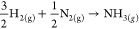
 and O_2_ is combined with H_2_ to produce H_2_O,^39,48^
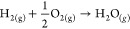
In those reactions, namely, ammonia synthesis and water formation, ε_N_2__ and ε_O_2__ can be isolated because both ammonia and water are generally well described by DFT, as they only contain N–H and O–H single bonds.^27^ Once the errors in O_2_ and N_2_ are corrected, ε_H_*__*x*__*_N_*__*y*__*_O_*__*z*__* can be calculated by combining eqs 2 and 4:
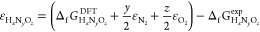
5

As will be shown in the subsequent section, there are ways of estimating ε_H_*__*x*__*_N_*__*y*__*_O_*__*z*__* based on the bonds and/or groups of atoms present in H_*x*_N_*y*_O_*z*_. The accuracy of the ε_H_*__*x*__*_N_*__*y*__*_O_*__*z*__* estimates will dictate the magnitude of the final errors (calculated with an updated version of eq 2), see below. In the following, we assume that ε_H_*__*x*__*_N_*__*y*__*_O_*__*z*__* is the sum of the errors due to the bonds and/or groups of atoms in H_*x*_N_*y*_O_*z*_. While this is usually a fair assumption, previous works^32^ showed that if a large functional group is present more than once in a small compound, additional intramolecular interactions might appear that change the magnitude of gas-phase corrections. In any case, it is possible to use the estimates of ε_H_*__*x*__*_N_*__*y*__*_O_*__*z*__* to correct the DFT-calculated Gibbs energy of formation as
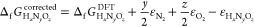
6

## Results and Discussion

**Figure 1:**
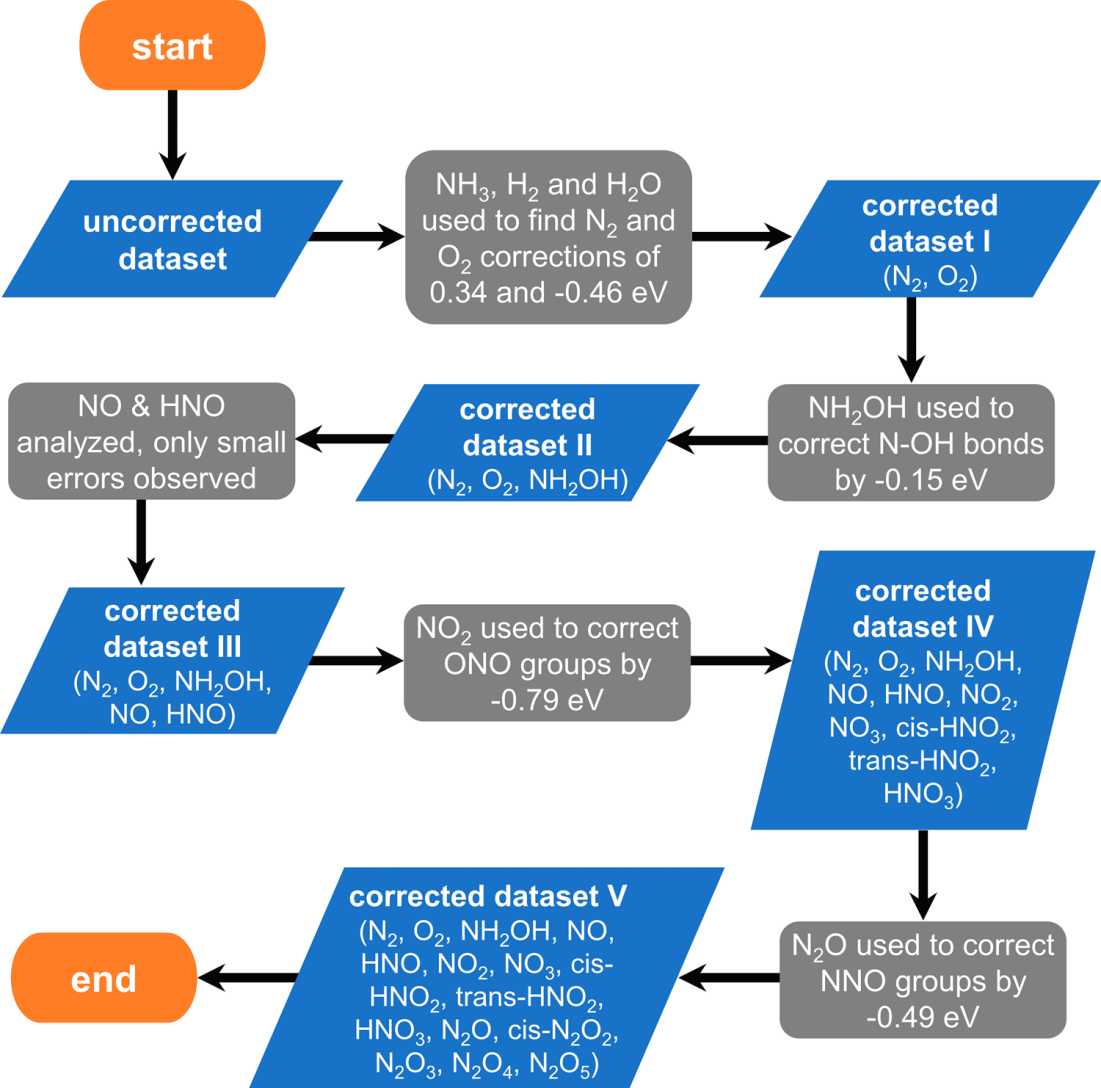
Flowchart of the sequential method used to correct the gas-phase formation energies of H_*x*_N_*y*_O_*z*_. Increasingly complex molecules are analyzed in every step until the entire dataset is corrected.

Once the errors have been isolated for each compound using eq 5, we employ a sequential correction approach (herein referred to as sequential) based on the complexity of the molecules (see the flowchart of the method in Figure 1). By complexity, we mean an increasing number of bonds and/or groups of atoms in the molecules. Initially, the simplest possible molecules are analyzed, and then increasingly large molecules are considered, to detect the possible bonds and/or functional groups responsible for the errors in their structures. In that order of ideas, the first compound we analyzed was hydroxylamine (NH_2_OH), as it only features single N–H, N–O and O–H bonds. The difference between the calculated and experimental formation energies is −0.15 eV, from which we conclude that single N–O bonds ought to be corrected by that much.

Next, we analyzed the N=O double bond present in nitric oxide (NO) and nitroxyl (HNO), the formation energies of which differ from experiments by 0.07 and 0.04 eV. As these errors are smaller than 0.10 eV, we opt not to correct them, although an additional average correction is an option if results with higher accuracy were needed. The next compound in the list is nitrogen dioxide (NO_2_), which displays a large error with respect to experiments of −0.79 eV. From this we conclude that the presence of an ONO backbone in a molecule induces an error of −0.79 eV in its calculated formation energy. Analogously, we note that the OCO backbone has been previously identified to induce appreciable errors in organic and inorganic C-containing compounds.^30,31^ The ONO backbone error together with the N–O single bond error allow us to correct the formation energies of nitrogen trioxide (NO_3_), *trans* and *cis* nitrous acid (*trans*-HNO_2_, *cis*-HNO_2_), and nitric acid (HNO_3_), which initially differ from experiments by as much as 1.38, 0.52, 0.52, and 0.94 eV. Upon the corrections, the residual errors are 0.19, 0.02, 0.02, and 0.00 eV.

The next compound in the list is nitrous oxide (N_2_O), the DFT formation energy of which initially differs from experiments by −0.49 eV. Therefore, we conclude that molecules with an NNO backbone have an error associated with it as large as −0.49 eV. In view of the lack of information, we assumed that errors in N–N bonds are one-half of those in the NNO backbone and the validity of this assumption will be asserted later in this work. Correcting the NNO, ONO, N–O and N–N errors, we are able to considerably lower the total errors in the formation energies from 0.83 to 0.10 eV for cis-N_2_O_2_, from 1.21 to 0.07 eV for N_2_O_3_, from 1.80 to 0.02 eV for N_2_O_4_, and from 1.98 to 0.11 eV for N_2_O_5_.

**Figure 2:**
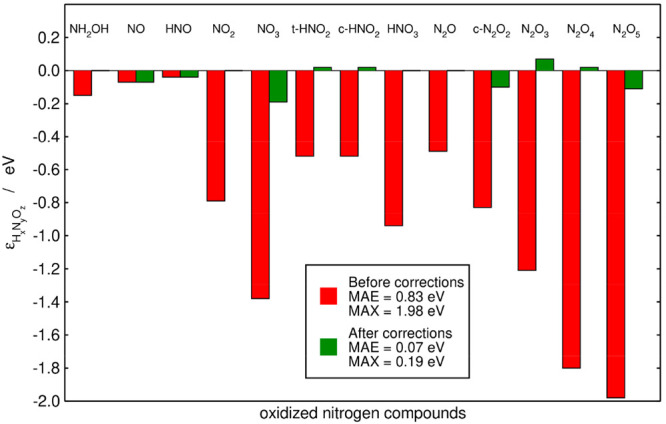
Initial (red) and final (green) errors in the nitrogen compounds under study. The final errors are obtained upon applying a sequential method that identifies N–OH and N–N bonds and ONO and NNO groups as sources of error. The mean and maximum absolute errors (MAE and MAX) are provided before and after the corrections in the inset. The experimental and final Gibbs energies are given in Table 1.

Overall, the mean and maximum absolute errors (MAE and MAX) are initially 0.83 and 1.98 eV. Once the N–O, N–N, ONO, and NNO errors have been corrected, the resulting MAE and MAX are drastically reduced to 0.07 and 0.19 eV. The initial and final errors for all compounds under study are provided in Figure 2.

A different approach consists of treating the problem as a mathematical optimization in which all errors are simultaneously lowered (hereon referred to as automated optimization 1, AO1). This strategy requires no chemical intuition to hierarchize the chemical compounds in increasing order of complexity, as in the sequential method. AO1 is a multiobjective optimization problem, where the MAE and MAX are minimized simultaneously (for further details see section S4 in the Supporting Information). The adjustable parameters for the optimization can be the errors in single N–O bonds, double N–O bonds, N–N bonds, and O–H bonds. A matrix can be built that decomposes every molecule into these bonds, such that the total error is a sum of all those contributions, see the representation of the 13 nitrogen compounds under study in section S3 in the Supporting Information.

**Figure 3:**
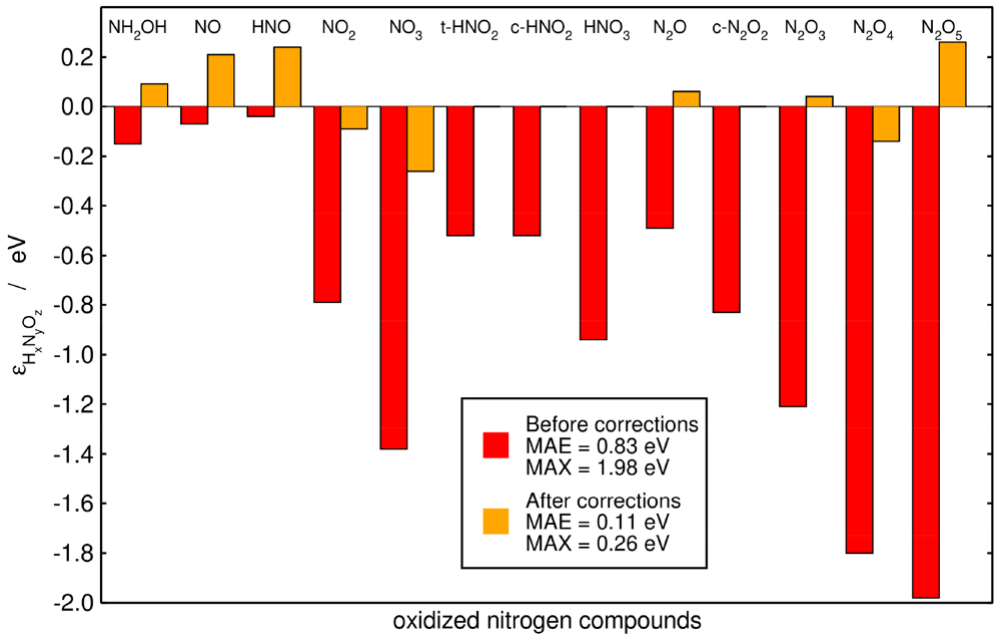
Initial (red) and final (orange) errors in the nitrogen compounds under study for AO1. The final errors are obtained upon minimizing simultaneously the MAE and MAX using as adjustable parameters the errors in N–O (single and double), N–N and O–H bonds. Inset: MAE and MAX before and after the corrections. The experimental and final Gibbs energies are given in Table 1.

In this case, there is no Pareto front as the MAE and MAX are minimized at the same point. The final MAE and MAX after AO1 are 0.11 and 0.26 eV. The initial and final errors upon this optimization are provided in Figure 3. Although this procedure substantially lowers the initial MAE and MAX, the sequential method in Figures 1 and 2 performs better (final MAEs: 0.07 vs 0.11 eV; final MAXs: 0.19 vs 0.26 eV).

**Figure 4:**
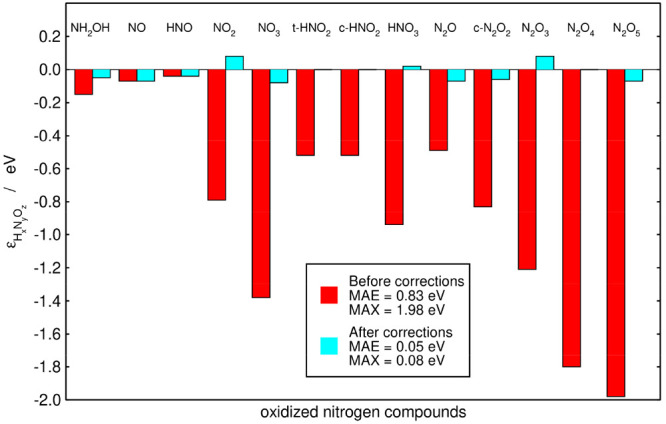
Initial (red) and final (cyan) errors in the nitrogen compounds under study for AO2. The final errors are obtained from the knee point of the simultaneous minimization of the MAE and MAX using as adjustable parameters the errors in N–O and N–N bonds and those in ONO and NNO groups. Inset: MAE and MAX values before and after the corrections. The experimental and final free energies are given in Table 1.

Similarly, one can capitalize on the errors found by the sequential method by using them as adjustable parameters for another optimization (hereon referred to as automated optimization 2, AO2), see sections S3 and S4. For this multiobjective optimization, the minimum distance selection method^49^ was used to find the most feasible point among the Pareto front, see section S4. The knee point or most satisfactory solution inside the feasible space corresponds to a MAE of 0.05 eV and a MAX of 0.08 eV. The initial and final errors obtained after this optimization are provided in Figure 4. The final MAE and MAX are visibly lower in Figure 4 (0.05 and 0.08 eV), compared to Figures 2 (0.07 and 0.19 eV) and 3 (0.11 and 0.26 eV), and a compromise between the magnitude of the MAX and MAE is attained. Section S5 in the Supporting Information also shows that AO2 is more accurate than a previous method based on the number of oxygen atoms in the molecules.

In Figures 2–4 we observe, in broad terms, that the larger the molecules, the larger the errors. In principle, this is because larger molecules have at least one problematic bond and/or group of atoms. However, this trend is not uniform neither in the initial nor the final errors, as the matrix representations of molecules that differ even by one oxygen atom can be rather different. For instance, N_2_O_4_ and N_2_O_5_ both have two ONO groups in the matrix representation of the sequential method, but the former has a NN bond while the latter has two NO single bonds (see Table S3). Within AO1, N_2_O_4_ and N_2_O_5_ both have two N=O bonds but the former has a N–N bond and two N–O bonds while the latter has four N–O bonds (see Table S4 in the Supporting Information).

**Figure 5:**
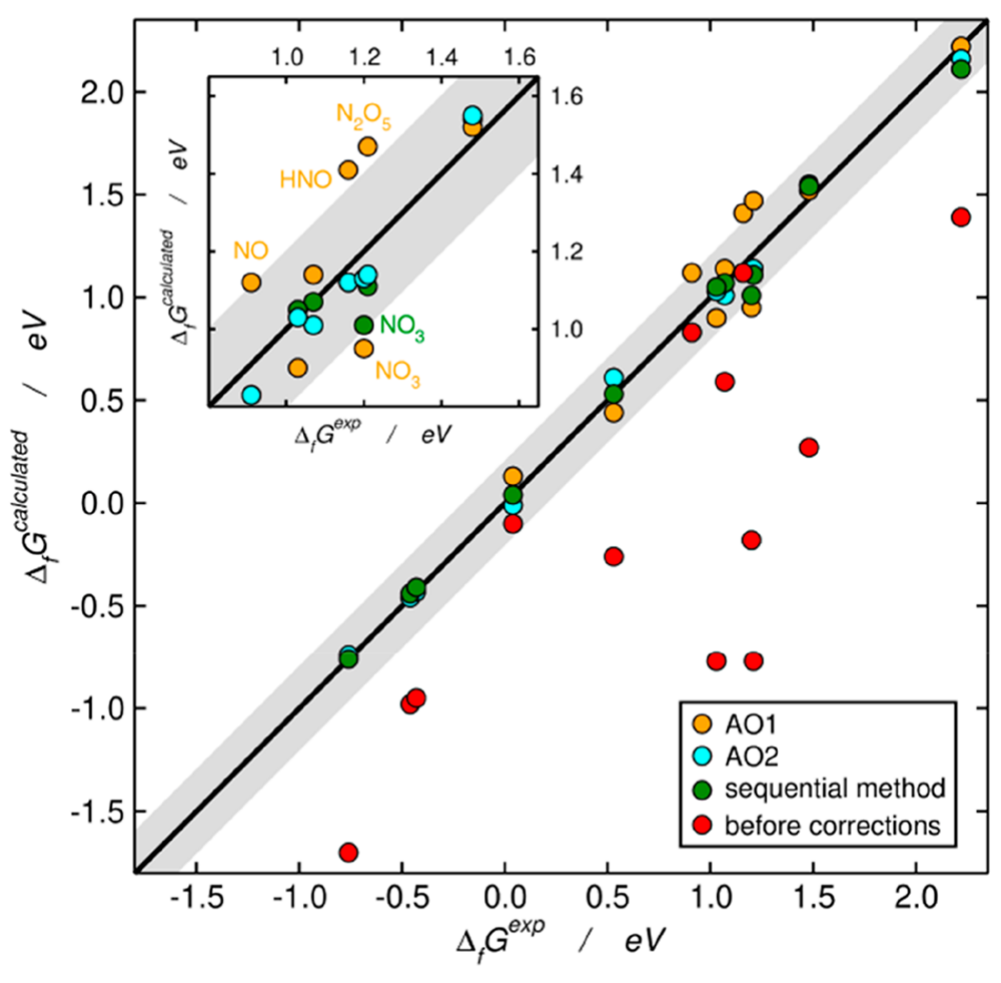
Parity plot for the formation energies of the nitrogen compounds under study. Red denotes data without corrections; green represents data corrected by the sequential method (Figure 2); orange denotes results from an automated optimization using as free parameters the errors in NO (single and double), NN and OH bonds (AO1, Figure 3); cyan represents the results from an automated optimization using as free parameters the errors in N–O and N–N bonds and in the ONO and NNO groups (AO2, Figure 4). Inset: region with the largest initial errors (Δ_f_*G*^exp^ from 0.80 to 1.65 eV). The gray band covers ±0.20 eV around the parity line.

Apart from visualizing the initial and final errors as in Figures 2–4, it is also convenient to analyze the formation energies to draw further conclusions. All formation energies are provided in Table 1, and Figure 5 presents a parity plot in which the experimental and calculated (using eq 5) formation energies are compared. The fact that the initial (red) data are below the parity line is explained in section S7 in the Supporting Information. The largest initial and final errors appear mostly in the range of 0.80 to 1.65 eV, which contains the lesser stable nitrogen compounds. *cis*-N_2_O_2_, which is the least-stable compound in this study, is an exception, because it displays large initial errors but small final errors. Importantly, the largest final errors in the sequential method and the two automated optimizations correspond to NO_3_. This suggests that the matrix representations of this compound might be somehow incomplete and/or that it might be advisible to assign a specific error to it if higher accuracy is needed.

**Table 1:** Formation Energies of Nitrogen Compounds^a^

	Formation Energies (eV)				
species	Δ_f_*G*_H_*x*_N_*y*_O_*z*__^exp^	Δ_f_*G*_H_*x*_N_*y*_O_*z*__^ONC^	Δ_f_*G*_H_*x*_N_*y*_O_*z*__^seq^	Δ_f_*G*_H_*x*_N_*y*_O_*z*__^AO1^	Δ_f_*G*_H_*x*_N_*y*_O_*z*__^AO2^
NH_2_OH	0.04	–0.11	0.04	0.13	–0.01
NO	0.91	0.83	0.83	1.12	0.83
HNO	1.16	1.12	1.12	1.41	1.12
NO_2_	0.53	–0.26	0.53	0.44	0.61
NO_3_	1.20	–0.18	1.01	0.95	1.13
*trans*-HNO_2_	–0.46	–0.98	–0.44	–0.46	–0.46
*cis*-HNO_2_	–0.43	–0.95	–0.41	–0.43	–0.43
HNO_3_	–0.76	–1.70	–0.76	–0.76	–0.74
N_2_O	1.07	0.59	1.07	1.14	1.01
*cis*-N_2_O_2_	2.22	1.39	2.11	2.22	2.16
N_2_O_3_	1.48	0.27	1.54	1.52	1.55
N_2_O_4_	1.03	–0.77	1.05	0.90	1.03
N_2_O_5_	1.21	–0.77	1.11	1.47	1.14

a Second column (energies denoted with a superscript “exp”) shows experimental formation energies; the third column (energies denoted with a superscript “ONC”) contains the DFT-calculated formation energies with O_2_ and N_2_ corrections. The fourth, fifth, and sixth columns give the final formation energies upon applying the sequential method in Figures 1 and 2 (energies denoted with a superscript “seq”) and automated optimizations 1 and 2 in Figures 3 and 4, respectively (energies denoted with superscripts “AO1” and “AO2”).

Table 2 shows the errors found by the three approaches for the bonds and groups of atoms within H_*x*_N_*y*_O_*z*_. The similarities between the values for the sequential method and AO2 are apparent and lead to three observations:

- Chemical intuition is able to detect and correct the errors to a great extent.
- Averaging among similar compounds leads to even greater accuracy, in particular in this case by lowering the MAX.
- Approximating the error in N–N bonds as one-half of the NNO error, as done in the sequential method, is the main difference with respect to AO2.

**Table 2:** Errors Present in Nitrogen Compounds, As Predicted by Three Different Approaches on the Basis of the Bonds and Groups of Atoms Present in the Molecules^a^

	Errors (eV)							
method	N–H	N–O	N=O	N–N	O–H	NOH	ONO	NNO
AO1	0.00	–0.42	–0.29	–0.26	0.18	–	–	–
AO2	0.00	–	–	–0.07	–	–0.09	–0.87	–0.42
sequential	0.00	–	–	–0.24	–	–0.15	–0.79	–0.49

a AO1 and AO2 are shown in Figures 3 and 4, and the sequential method is shown in Figures 1 and 2. Further details appear in sections S3 and S4 in the Supporting Information.

Based on these observations, we first averaged the errors of the sequential method for NO_2_ and NO_3_ to assess the ONO error. Second, N_2_H_4_, which should only have an error in its N–N bond, was calculated in a previous work.^32^ The error of −0.09 eV reported in that work is close to that found by AO2 (−0.07 eV, see Table 2). With these two amends, the MAE and MAX of the sequential method are lowered to 0.06 and 0.13 eV.

Furthermore, some bond errors in AO1 can be combined to approximate the group errors found by the sequential method. For instance, summing the errors of N–O and N=O bonds gives −0.70 eV, which is close to the value of −0.79 eV obtained by the sequential method for the ONO group. Besides, the sum of the N=O and N–N bonds found with AO1 is −0.55 eV, which is also close to the value obtained by the sequential method for the NNO group (−0.49 eV). The same holds for the NOH group: from the addition of the N–O and O–H errors we get −0.23 eV, while the sequential method finds −0.15 eV. Lastly, the N–N errors between the sequential method and AO1 are also rather close (−0.24 and −0.26 eV). However, we stress that it is the small discrepancies between the methods that are ultimately responsible for the different final MAEs and MAXs observed in Figures 2–4. We also note that section S5 in the Supporting Information verifies that N–H bonds are generally well described and expands on the error in O–H bonds found by AO1.

Before closing the discussion, we note that, when the decomposition of a given molecule into its groups is not unambiguous, running several tests is advisible to determine the best representation. For instance, *trans*-HNO_2_ in the sequential method can be thought of having an ONO group and an OH bond. This leads to a residual error of 0.35 eV. In contrast, considering it to be composed of 0.5 ONO groups and a single NO bond, the residual error is 0.00 eV. The latter representation can also be used for *cis*-HNO_2_ and extended to HNO_3_. Moreover, *cis*-N_2_O_2_ can be represented as two NO units linked by a NN bond, leading to a residual error of 0.76 eV. When represented as two NNO units and the double-counting of the NN bond is discounted, the residual error is 0.06 eV.

## Impact on Heterogeneous (Electro)catalysis

By correcting gas-phase errors, it is possible to obtain without relying on fortuitous error cancellation accurate reaction energies, equilibrium potentials and, in principle, adsorption energies. This is critical for the models used in computational heterogeneous (electro)catalysis, which are usually based on these properties.

For example, it was shown in a recent work for electrochemical ammonia synthesis and electrochemical nitric oxide reduction to hydroxylamine that gas-phase corrections modify the predicted overpotentials, Sabatier-type volcano plots, and the ordering of catalytic activities among the analyzed materials.^34^ In addition, gas-phase corrections have also been shown to improve the prediction of equilibrium and onset potentials for the electroreduction of CO_2_ to CO,^30^ and brought DFT-calculated adsorption energies of CO on several metals closer to experimental values.^50^ Finally, the importance of gas-phase corrections has also been illustrated for free-energy diagrams and volcano plots for O_2_ reduction and evolution^39,51^ and H_2_O_2_ production.^40^

Furthermore, if a given compound X participates in a catalytic reaction but no experimental data are available for it, one can decompose it in its bonds and/or groups of atoms and anticipate the errors present in its DFT-calculated free energy of formation. However, it is recommendable to make an ensemble of predictions based on different matrix representations and establish a correction range rather than a specific correction.

Finally, we believe that the proposed approaches could be transferred to assess the errors of adsorbates at surfaces. Given their semiempirical nature, this extension would presuppose the availability of accurate experimental adsorption energies.

## Conclusions

Herein, we showed that large errors are found when using PBE to assess the free energies of formation of 13 gaseous compounds containing nitrogen, hydrogen, and oxygen. To identify and reduce such errors, we proposed approaches based on chemical intuition and a matrix representation of the molecules. The representations decompose each molecule into the bonds and/or groups of atoms they contain. We considered three methods: a sequential method based on the analysis of increasingly complex molecules, an automated optimization method based on the bonds present in the molecules, and an automated optimization method based on the findings of the sequential method. The sequential method identified single N–O and N–N bonds together with NNO and ONO backbones as the largest error sources. On the other hand, the automated optimization method based on bonds deemed N–O, N=O, N–N, and O–H bonds as being problematic.

Comparison of the MAEs and MAXs among the three approaches indicates that the bond optimization method is the least accurate, while the optimization based on the errors detected sequentially is the best. This shows that (i) chemical intuition can be used to boost automated routines for error minimization, and (ii) the accuracy of the PBE functional to predict the thermochemistry of nitrogen compounds can be semiempirically enhanced, bypassing the need for more expensive levels of theory. Finally, we emphasize that the analysis shown here was made for PBE and nitrogen compounds but can be easily extended to other functionals and families of compounds.

## Supporting Information Available

Schematics of the nitrogen compounds under study, tabulated data, cutoff convergence tests, matrix representations of the nitrogen compounds, details of the optimization procedure, additional tests and comparison to previous works, enthalpic effects, analysis of Figure 5, and direct coordinates of the converged calculations (PDF)

## References

[ref1] Rosca V.; Duca M.; de Groot M. T.; Koper M. T. M. Nitrogen Cycle Electrocatalysis. Chem. Rev. 2009, 109 (6), 2209–2244. 10.1021/cr8003696. 19438198

[ref2] Schlesinger W. H.; Reckhow K. H.; Bernhardt E. S. Global Change: The Nitrogen Cycle and Rivers. Water Resour. Res. 2006, 42 (3), W03S06 10.1029/2005WR004300.

[ref3] Rivett M. O.; Buss S. R.; Morgan P.; Smith J. W. N.; Bemment C. D. Nitrate Attenuation in Groundwater: A Review of Biogeochemical Controlling Processes. Water Res. 2008, 42 (16), 4215–4232. 10.1016/j.watres.2008.07.020. 18721996

[ref4] Aneja V. P.; Schlesinger W. H.; Erisman J. W. Farming Pollution. Nat. Geosci. 2008, 1 (7), 409–411. 10.1038/ngeo236.

[ref5] Sutton M. A.; Bleeker A. The Shape of Nitrogen to Come. Nature 2013, 494 (7438), 435–437. 10.1038/nature11954. 23426258

[ref6] Wuebbles D. J. Nitrous Oxide: No Laughing Matter. Science 2009, 326 (5949), 56–57. 10.1126/science.1179571. 19797649

[ref7] Rockström J.; Steffen W.; Noone K.; Persson Å.; Chapin F. S.; Lambin E. F.; Lenton T. M.; Scheffer M.; Folke C.; Schellnhuber H. J.; Nykvist B.; de Wit C. A.; Hughes T.; van der Leeuw S.; Rodhe H.; Sörlin S.; Snyder P. K.; Costanza R.; Svedin U.; Falkenmark M.; Karlberg L.; Corell R. W.; Fabry V. J.; Hansen J.; Walker B.; Liverman D.; Richardson K.; Crutzen P.; Foley J. A. A Safe Operating Space for Humanity. Nature 2009, 461 (7263), 472–475. 10.1038/461472a. 19779433

[ref8] Galloway J. N.; Townsend A. R.; Erisman J. W.; Bekunda M.; Cai Z.; Freney J. R.; Martinelli L. A.; Seitzinger S. P.; Sutton M. A. Transformation of the Nitrogen Cycle: Recent Trends, Questions, and Potential Solutions. Science 2008, 320 (5878), 889–892. 10.1126/science.1136674. 18487183

[ref9] Galloway J. N.; Aber J. D.; Erisman J. W.; Seitzinger S. P.; Howarth R. W.; Cowling E. B.; Cosby B. J. The Nitrogen Cascade. BioScience 2003, 53 (4), 341–356. 10.1641/0006-3568(2003)053[0341:TNC]2.0.CO;2.

[ref10] Kampa M.; Castanas E. Human Health Effects of Air Pollution. Environ. Pollut. 2008, 151 (2), 362–367. 10.1016/j.envpol.2007.06.012. 17646040

[ref11] Hakeem K. R.; Sabir M.; Ozturk M.; Akhtar Mohd. S.; Ibrahim F. H. Nitrate and Nitrogen Oxides: Sources, Health Effects and Their Remediation. In Reviews of Environmental Contamination and Toxicology, Vol. 242; de Voogt P., Ed.; Springer International Publishing, 2017; pp 183–217, 10.1007/398_2016_11. 27734212

[ref12] Jones K. The Chemistry of Nitrogen, Vol. 11; Pergamon: Oxford, U.K., 1973, 10.1016/C2013-0-05694-0.

[ref13] Glendening E. D.; Halpern A. M. Ab Initio Calculations of Nitrogen Oxide Reactions: Formation of N_2_O_2_, N_2_O_3_, N_2_O_4_, N_2_O_5_, and N_4_O_2_ from NO, NO_2_, NO_3_, and N_2_O. J. Chem. Phys. 2007, 127 (16), 164307 10.1063/1.2777145. 17979338

[ref14] Aplincourt P.; Bohr F.; Ruiz-Lopez M. F. Density Functional Studies of Compounds Involved in Atmospheric Chemistry: Nitrogen Oxides. J. Mol. Struct. THEOCHEM 1998, 426 (1–3), 95–104. 10.1016/S0166-1280(97)00311-4.

[ref15] Stirling A.; Pápai I.; Mink J.; Salahub D. R. Density Functional Study of Nitrogen Oxides. J. Chem. Phys. 1994, 100 (4), 2910–2923. 10.1063/1.466433.

[ref16] Jursic B. S. A Study of Nitrogen Oxides by Using Density Functional Theory and Their Comparison with Ab Initio and Experimental Data. Int. J. Quantum Chem. 1996, 58 (1), 41–46. 10.1002/(SICI)1097-461X(1996)58:1<41::AID-QUA5>3.0.CO;2-Y.

[ref17] Jitariu L. C.; Hirst D. M. Theoretical Investigation of the N_2_O_5_ ⇌ NO_2_ + NO_3_ Equilibrium by Density Functional Theory and Ab Initio Calculations. Phys. Chem. Chem. Phys. 2000, 2 (4), 847–852. 10.1039/a906864c.

[ref18] Janoschek R.; Kalcher J. The NO_3_ Radical and Related Nitrogen Oxides, Characterized by Ab Initio Calculations of Thermochemical Properties. Z. Für Anorg. Allg. Chem. 2002, 628 (12), 2724–2730. 10.1002/1521-3749(200212)628:12<2724::AID-ZAAC2724>3.0.CO;2-E.

[ref19] Becke A. D. Density-functional Thermochemistry. III. The Role of Exact Exchange. J. Chem. Phys. 1993, 98 (7), 5648–5652. 10.1063/1.464913.

[ref20] Paier J.; Marsman M.; Kresse G. Why Does the B3LYP Hybrid Functional Fail for Metals?. J. Chem. Phys. 2007, 127 (2), 024103 10.1063/1.2747249. 17640115

[ref21] Marsman M.; Paier J.; Stroppa A.; Kresse G. Hybrid Functionals Applied to Extended Systems. J. Phys.: Condens. Matter 2008, 20 (6), 064201 10.1088/0953-8984/20/6/064201. 21693863

[ref22] Seh Z. W.; Kibsgaard J.; Dickens C. F.; Chorkendorff I.; Nørskov J. K.; Jaramillo T. F. Combining Theory and Experiment in Electrocatalysis: Insights into Materials Design. Science 2017, 355 (6321), eaad4998 10.1126/science.aad4998. 28082532

[ref23] Janthon P.; Luo S. A.; Kozlov S. M.; Viñes F.; Limtrakul J.; Truhlar D. G.; Illas F. Bulk Properties of Transition Metals: A Challenge for the Design of Universal Density Functionals. J. Chem. Theory Comput. 2014, 10 (9), 3832–3839. 10.1021/ct500532v. 26588528

[ref24] Ropo M.; Kokko K.; Vitos L. Assessing the Perdew-Burke-Ernzerhof Exchange-Correlation Density Functional Revised for Metallic Bulk and Surface Systems. Phys. Rev. B 2008, 77 (19), 195445 10.1103/PhysRevB.77.195445.

[ref25] Janthon P.; Kozlov S. M.; Viñes F.; Limtrakul J.; Illas F. Establishing the Accuracy of Broadly Used Density Functionals in Describing Bulk Properties of Transition Metals. J. Chem. Theory Comput. 2013, 9 (3), 1631–1640. 10.1021/ct3010326. 26587624

[ref26] Vega L.; Ruvireta J.; Viñes F.; Illas F. Jacob’s Ladder as Sketched by Escher: Assessing the Performance of Broadly Used Density Functionals on Transition Metal Surface Properties. J. Chem. Theory Comput. 2018, 14 (1), 395–403. 10.1021/acs.jctc.7b01047. 29182868

[ref27] Kurth S.; Perdew J. P.; Blaha P. Molecular and Solid-State Tests of Density Functional Approximations: LSD, GGAs, and Meta-GGAs. Int. J. Quantum Chem. 1999, 75 (4–5), 889–909. 10.1002/(SICI)1097-461X(1999)75:4/5<889::AID-QUA54>3.0.CO;2-8.

[ref28] Ernzerhof M.; Scuseria G. E. Assessment of the Perdew–Burke–Ernzerhof Exchange-Correlation Functional. J. Chem. Phys. 1999, 110 (11), 5029–5036. 10.1063/1.478401. 15268348

[ref29] Tao J.; Perdew J. P.; Staroverov V. N.; Scuseria G. E. Climbing the Density Functional Ladder: Nonempirical Meta--Generalized Gradient Approximation Designed for Molecules and Solids. Phys. Rev. Lett. 2003, 91 (14), 146401 10.1103/PhysRevLett.91.146401. 14611541

[ref30] Granda-Marulanda L. P.; Rendón-Calle A.; Builes S.; Illas F.; Koper M. T. M.; Calle-Vallejo F. A Semiempirical Method to Detect and Correct DFT-Based Gas-Phase Errors and Its Application in Electrocatalysis. ACS Catal. 2020, 10 (12), 6900–6907. 10.1021/acscatal.0c01075.

[ref31] Peterson A. A.; Abild-Pedersen F.; Studt F.; Rossmeisl J.; Nørskov J. K. How Copper Catalyzes the Electroreduction of Carbon Dioxide into Hydrocarbon Fuels. Energy Environ. Sci. 2010, 3 (9), 1311–1315. 10.1039/c0ee00071j.

[ref32] Urrego-Ortiz R.; Builes S.; Calle-Vallejo F. Fast Correction of Errors in the DFT-Calculated Energies of Gaseous Nitrogen-Containing Species. ChemCatChem. 2021, 13 (10), 2508–2516. 10.1002/cctc.202100125.

[ref33] Christensen R.; Hansen H. A.; Vegge T. Identifying Systematic DFT Errors in Catalytic Reactions. Catal. Sci. Technol. 2015, 5 (11), 4946–4949. 10.1039/C5CY01332A.

[ref34] Urrego-Ortiz R.; Builes S.; Calle-Vallejo F. Impact of Intrinsic Density Functional Theory Errors on the Predictive Power of Nitrogen Cycle Electrocatalysis Models. ACS Catal. 2022, 12 (8), 4784–4791. 10.1021/acscatal.1c05333. 35465243 PMC9017217

[ref35] Ernzerhof M.; Perdew J. P.; Burke K. Coupling-Constant Dependence of Atomization Energies. Int. J. Quantum Chem. 1997, 64 (3), 285–295. 10.1002/(SICI)1097-461X(1997)64:3<285::AID-QUA2>3.0.CO;2-S.

[ref36] Perdew J. P.; Burke K.; Ernzerhof M. Generalized Gradient Approximation Made Simple. Phys. Rev. Lett. 1996, 77 (18), 3865–3868. 10.1103/PhysRevLett.77.3865. 10062328

[ref37] Kresse G.; Furthmüller J. Efficient Iterative Schemes for Ab Initio Total-Energy Calculations Using a Plane-Wave Basis Set. Phys. Rev. B 1996, 54 (16), 11169–11186. 10.1103/PhysRevB.54.11169. 9984901

[ref38] Kresse G.; Joubert D. From Ultrasoft Pseudopotentials to the Projector Augmented-Wave Method. Phys. Rev. B 1999, 59 (3), 1758–1775. 10.1103/PhysRevB.59.1758.

[ref39] Sargeant E.; Illas F.; Rodríguez P.; Calle-Vallejo F. Importance of the Gas-Phase Error Correction for O_2_ When Using DFT to Model the Oxygen Reduction and Evolution Reactions. J. Electroanal. Chem. 2021, 896, 115178 10.1016/j.jelechem.2021.115178.

[ref40] Almeida M. O.; Kolb M. J.; Lanza M. R. V.; Illas F.; Calle-Vallejo F. Gas-Phase Errors Affect DFT-Based Electrocatalysis Models of Oxygen Reduction to Hydrogen Peroxide. ChemElectroChem. 2022, 9 (12), e20220021 10.1002/celc.202200210.

[ref41] Misener R.; Floudas C. A. ANTIGONE: Algorithms for CoNTinuous/Integer Global Optimization of Nonlinear Equations. J. Glob. Optim. 2014, 59 (2–3), 503–526. 10.1007/s10898-014-0166-2.

[ref42] Czyzyk J.; Mesnier M. P.; More J. J. The NEOS Server. IEEE Comput. Sci. Eng. 1998, 5 (3), 68–75. 10.1109/99.714603.

[ref43] Gropp W.; More J. J. Optimization environments and the NEOS server. Available via the Internet at: https://www.osti.gov/biblio/563264 (accessed May 14. 2022).

[ref44] Haynes W. M.; Lide D. R.; Bruno T. J. CRC Handbook of Chemistry and Physics, 97th Edition; CRC Press/Taylor and Francis:: Boca Raton, FL, 2016, 10.1201/9781315380476.

[ref45] Linstrom P. J.; Mallard W. G. NIST Chemistry WebBook, NIST Standard Reference Database No. 69; National Institute of Standards and Technology: Gaithersburg, MD, 2022, 10.18434/T4D303.

[ref46] Bartel C. J.; Weimer A. W.; Lany S.; Musgrave C. B.; Holder A. M. The Role of Decomposition Reactions in Assessing First-Principles Predictions of Solid Stability. Npj Comput. Mater. 2019, 5 (1), 4 10.1038/s41524-018-0143-2.

[ref47] Nørskov J. K.; Rossmeisl J.; Logadottir A.; Lindqvist L.; Kitchin J. R.; Bligaard T.; Jónsson H. Origin of the Overpotential for Oxygen Reduction at a Fuel-Cell Cathode. J. Phys. Chem. B 2004, 108 (46), 17886–17892. 10.1021/jp047349j. 39682080

[ref48] Calle-Vallejo F.; Martínez J. I.; García-Lastra J. M.; Mogensen M.; Rossmeisl J. Trends in Stability of Perovskite Oxides. Angew. Chem., Int. Ed. 2010, 49 (42), 7699–7701. 10.1002/anie.201002301. 20836100

[ref49] Sun G.; Li G.; Zhou S.; Li H.; Hou S.; Li Q. Crashworthiness Design of Vehicle by Using Multiobjective Robust Optimization. Struct. Multidiscip. Optim. 2011, 44 (1), 99–110. 10.1007/s00158-010-0601-z.

[ref50] Wellendorff J.; Silbaugh T. L.; Garcia-Pintos D.; Nørskov J. K.; Bligaard T.; Studt F.; Campbell C. T. A Benchmark Database for Adsorption Bond Energies to Transition Metal Surfaces and Comparison to Selected DFT Functionals. Surf. Sci. 2015, 640, 36–44. 10.1016/j.susc.2015.03.023.

[ref51] Sargeant E.; Illas F.; Rodríguez P.; Calle-Vallejo F. On the Shifting Peak of Volcano Plots for Oxygen Reduction and Evolution. Electrochim. Acta 2022, 426, 140799 10.1016/j.electacta.2022.140799.

