# Supplementary material for: Automated versus Chemically Intuitive Deconvolution of Density Functional Theory (DFT)-Based Gas-Phase Errors in Nitrogen Compounds
Source: Ind Eng Chem Res. 2022 Sep 2;61(36):13375–82. doi: 10.1021/acs.iecr.2c02111 (PMC9479071; doi:10.1021/acs.iecr.2c02111)
Supplement: Supplementary file 1 — ie2c02111_si_001.pdf [file ie2c02111_si_001.pdf]

Supporting Information

Automated versus chemically intuitive deconvolution of DFT-based gas-phase errors in nitrogen compounds

Ricardo Urrego-Ortiz,[1] Santiago Builes,[1,*] Federico Calle-Vallejo[2,3,4,*]

[1] Departamento de Ingeniería de Procesos, Universidad EAFIT, Carrera 49 # 7 sur 50, 050022, Medellín, Colombia.

[2] Department of Materials Science and Chemical Physics & Institute of Theoretical and Computational Chemistry, University of Barcelona, Martí i Franquès 1, 08028 Barcelona, Spain.

[3] Nano-Bio Spectroscopy Group and European Theoretical Spectroscopy Facility (ETSF), Department of Polymers and Advanced Materials: Physics, Chemistry and Technology, University of the Basque Country UPV/EHU, Avenida Tolosa 72, 20018 San Sebastián, Spain.

[4] IKERBASQUE, Basque Foundation for Science, Plaza de Euskadi 5, 48009 Bilbao, Spain.

*Correspondence to: sbuiles@eafit.edu.co, f.calle.vallejo@ub.edu

Table of contents

S1. Schematics of the nitrogen compounds under study 2

S2. Tabulated data and convergence tests 2

S3. Matrix representations of the nitrogen compounds 4

S4. Optimization procedure 5

S5. Free and fixed parameter tests and comparison to previous works 6

S6. Enthalpic effects 9

S7. Analysis of the initial trends in Figure 5 10

S8. Direct coordinates 10

References 11

S1. Schematics of the nitrogen compounds

Figure S1 shows the ball-and-stick representations, the names and molecular formulas of the 13 nitrogen compounds under study.

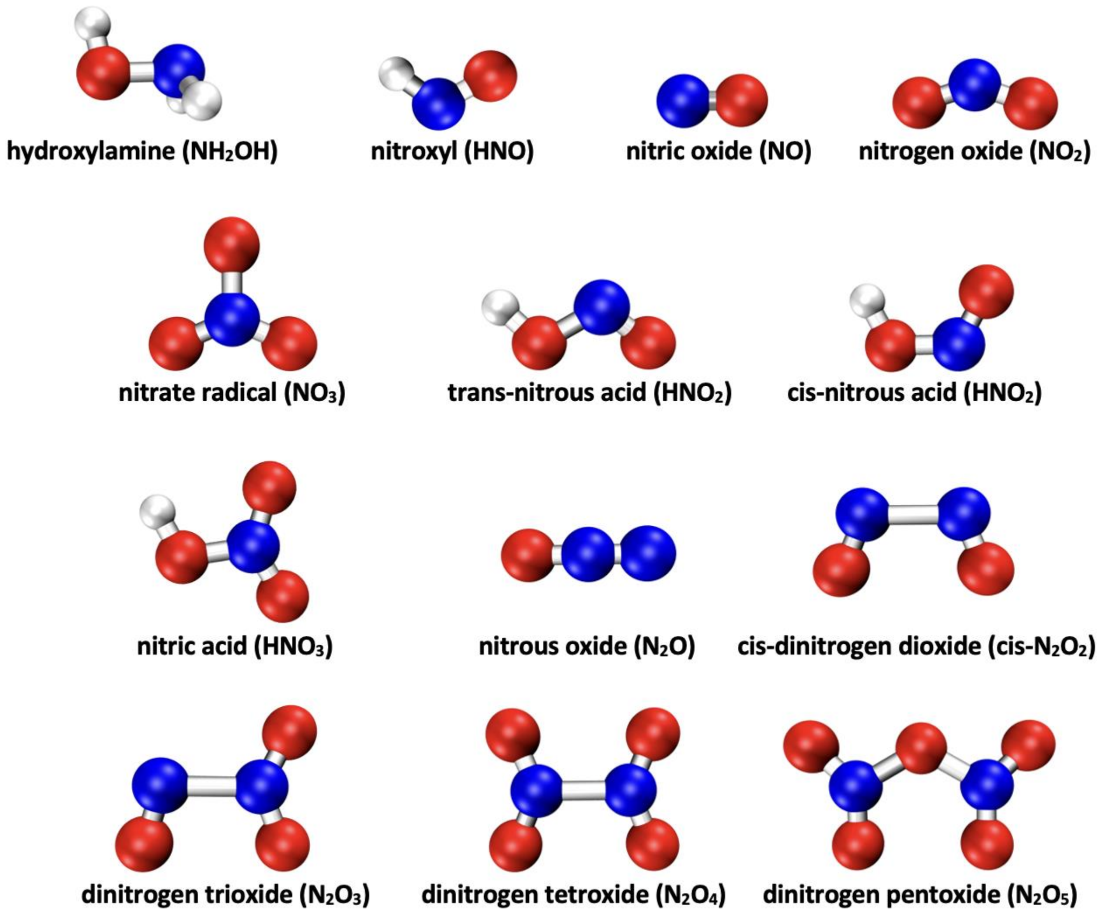

Figure S1. Ball-and-stick representation of the nitrogen compounds in this study. Blue balls correspond to nitrogen atoms, red balls to oxygen atoms and white balls to hydrogen atoms.

S2. Tabulated data and convergence tests

Table S1 shows the DFT-calculated zero-point energies (ZPEs) and TS corrections at T = 298.15 K for the 13 compounds under study together with the values for H_2, O_2, N_2, H_2O and NH_3, which are necessary for the assessment of the errors.

Figure S2 shows that the formation energies of N_2O are converged at a plane-wave cutoff of 450 eV. The convergence of the ZPEs is illustrated in Table S2.

Table S1. DFT-calculated ZPE and experimental[1,2] TS corrections for the compounds in this study at 298.15 K. All values are in eV and were calculated with PBE.

species	ZPE	TS
H_2	0.27	0.40
O_2	0.10	0.63
N_2	0.15	0.59
H_2O	0.57	0.58
NH_3	0.91	0.60
NH_2OH	1.06	0.73
NO	0.12	0.65
HNO	0.37	0.68
NO_2	0.23	0.74
NO_3	0.35	0.78
trans-HNO_2	0.53	0.77
cis-HNO_2	0.53	0.77
HNO_3	0.69	0.82
N_2O	0.30	0.68
cis-N_2O_2	0.31	0.92
N_2O_3	0.45	0.97
N_2O_4	0.61	0.94
N_2O_5	0.71	1.07

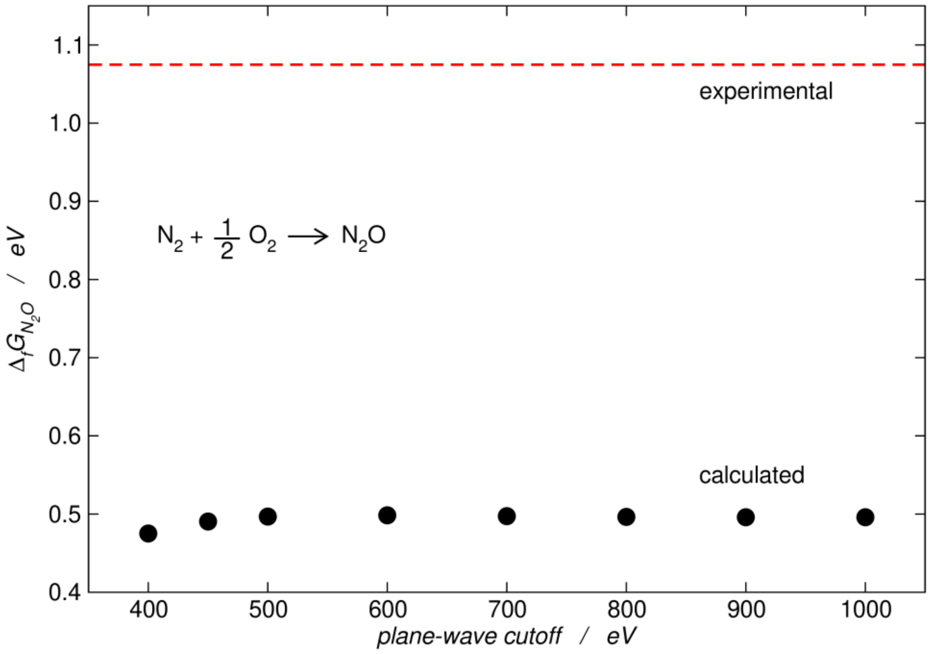

Figure S2. Formation energy of N_2O at various cutoffs. The red line is the experimental value (1.07 eV).

Table S2. Convergence of the zero-point energies. All values are in eV.

Plane-wave cutoff	ZPE_{O2}	ZPE_{N2}	ZPE_{N2O}	ΔZPE
400	0.10	0.16	0.30	0.10
700	0.10	0.15	0.30	0.10
1000	0.10	0.15	0.30	0.10

S3. Matrix representations of the nitrogen compounds

Table S3 contains the matrix representation of the 13 compounds under study for the sequential method and AO2. A negative number is used for cis-N_2O_2 to avoid the double counting of its NN bond.

Table S3. Matrix representation of nitrogen compounds according to the sequential method and AO2.

species	ONO	NNO	NOH	NN
NH_2OH	0.0	0.0	1.0	0.0
NO	0.0	0.0	0.0	0.0
HNO	0.0	0.0	0.0	0.0
NO_2	1.0	0.0	0.0	0.0
NO_3	1.5	0.0	0.0	0.0
trans-HNO_2	0.5	0.0	1.0	0.0
cis-HNO_2	0.5	0.0	1.0	0.0
HNO_3	1.0	0.0	1.0	0.0
N_2O	0.0	1.0	0.0	0.0
cis-N_2O_2	0.0	2.0	0.0	-1.0
N_2O_3	1.0	1.0	0.0	0.0
N_2O_4	2.0	0.0	0.0	1.0
N_2O_5	2.0	0.0	2.0	0.0

Table S4 contains the matrix representation of the nitrogen-containing compounds for AO1.

Table S4. Matrix representation of nitrogen compounds according to AO1.

species	N-O	N=O	N-N	O-H
NH_2OH	1	0	0	1
NO	0	1	0	0
HNO	0	1	0	0
NO_2	1	1	0	0
NO_3	2	1	0	0
trans-HNO_2	1	1	0	1
cis-HNO_2	1	1	0	1
HNO_3	2	1	0	1
N_2O	0	1	1	0
cis-N_2O_2	0	2	1	0
N_2O_3	1	2	1	0
N_2O_4	2	2	1	0
N_2O_5	4	2	0	0

S4. Optimization procedure

The nonlinear programming (NLP) optimization problem was formulated as a minimization of the objective functions:

$$MAE = abs\left(\frac{1}{n}\sum_{i=1}^{n}\left(\Delta_f G_{\mathrm{i}}^{corrected} - \Delta_f G_{\mathrm{i}}^{exp}\right)\right) \tag{S1}$$

$$MAX = max\left(abs\left(\Delta_f G_{\mathrm{i}}^{corrected} - \Delta_f G_{\mathrm{i}}^{exp}\right)\right) \tag{S2}$$

where abs() is the absolute value of the function, max() is the maximum value of the function and $\Delta_f G_{\mathrm{i}}^{corrected} - \Delta_f G_{\mathrm{i}}^{exp}$ is the absolute difference between the experimental formation Gibbs energy and the DFT corrected value for each substance i. A reformulation of the abs() and max() functions was used to avoid the use of non-smooth functions in the optimization.[3] The absolute value was split into the sum of its positive and negative parts, each represented by a positive variable. Thus, the discontinuity in the derivative of the function abs() is converted into additional restrictions to the optimization. The max function was replaced by 13 inequalities (one for each nitrogen compound) such that the feasible space was enlarged but the discontinuity of the function was removed.

The multi-objective optimization problem formulated for AO1 found that the utopia point was within the feasible space. In other words, the minimum possible MAE

corresponds to the minimum possible MAX. However, for AO2 the utopia point was not inside the feasible space and a Pareto front was obtained, see Figure S3.

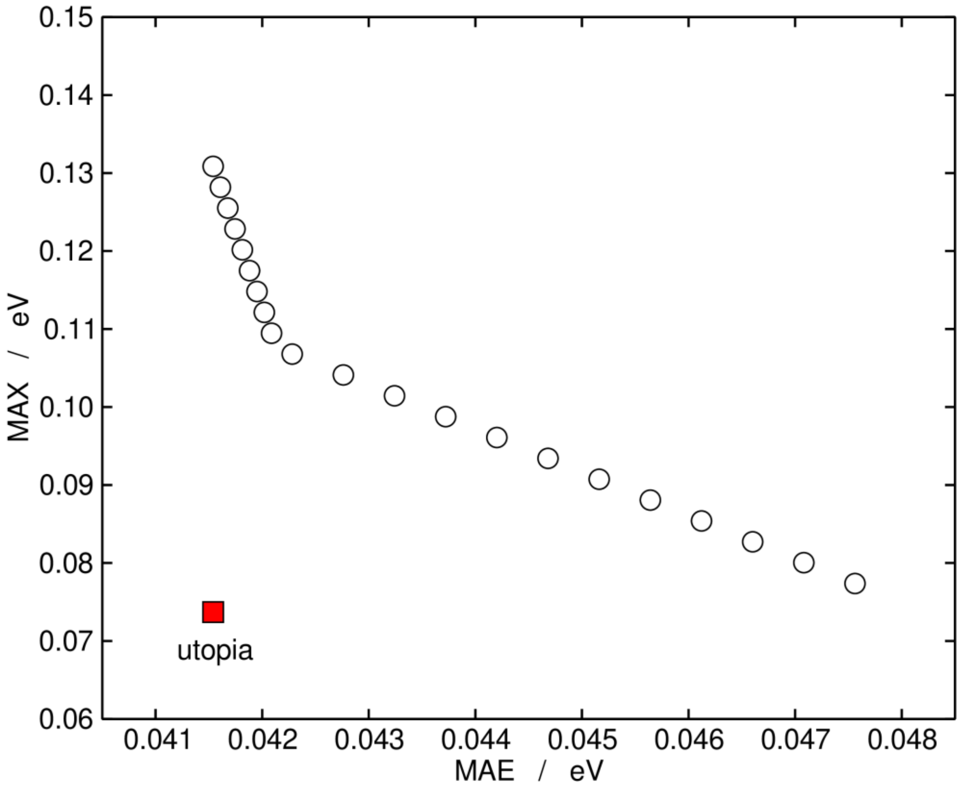

Figure S3. Pareto front (white circles) and utopia point (red square) for AO2.

S5. Free and fixed parameter tests and comparison to previous works

In general, it is assumed that O-H bonds are reasonably well described, but this might vary from one family of compounds to the next. AO1 is not biased by design to assume a good description of O-H bonds, but one could easily assume that error to be zero and reoptimize with it as a fixed parameter. In doing so, via AO1 we obtained a slightly larger MAE and nearly the same values for the rest of the free parameters, see Table S5. While this result calls for a thorough parameter sensitivity analysis that escapes our current scope, it suggests that there is a hierarchy of parameters in AO1 in which O-H bonds are not as important other bonds.

Table S5. Errors for AO1 obtained by setting the error in O-H bonds to zero (middle column) and by using it as a free parameter (right column).

bond error	results with fixed OH / eV	original results / eV
N-H	0.00	0.00
N-O	-0.41	-0.42
N=O	-0.30	-0.29
N-N	-0.23	-0.22
O-H	0.00	0.18
MAE	0.16	0.11
MAX	0.26	0.26

In addition, we set the errors in NH bonds as a free parameter in AO2 for the Pareto optimal point. Table S6 shows that the NH error is nearly zero, while the MAE, MAX and the other group errors are similar. This suggests that NH bonds are indeed generally well described.

Table S6. Errors for AO2 obtained by allowing the error in N-H bonds to be a free parameter (middle column) and by fixing it to be zero (right column).

bond & group errors	results with free NH / eV	original results / eV
NH	-0.02	0.00
ONO	-0.87	-0.87
NNO	-0.42	-0.42
NOH	-0.09	-0.12
N-N	-0.01	-0.01
MAE	0.04	0.05
MAX	0.08	0.08

In an earlier work[4] we used the number of oxygen atoms (n_o) as a descriptor to predict the errors in the DFT-energies of 11 gaseous nitrogen compounds split in three groups, namely NO_x, N_2O_X and HNO_x. The rationale behind this descriptor is that the progressive addition of oxygen atoms leads to the formation of multiple bonds, which are poorly described by GGAs.[5] Such correction scheme is based on Equation S3:

$$\Delta_f H^{corr}_{H_xN_yO_z} = \left(\Delta_f H^{DFT}_{H_xN_yO_z} + \frac{y}{2}\varepsilon_{N_2} + \frac{z}{2}\varepsilon_{O_2}\right) - m_i \cdot n_O - b_i \qquad \text{(S3)}$$

Table S7 shows the errors in ε_{N_2} and ε_{O_2}, together with the parameters m_i and b_i for NO_x, N_2O_x, and HNO_x, for the PBE exchange-correlation functional.

Table S7. Parameters for the correction of errors using as a descriptor the number of oxygen atoms in the molecules. ε_{O_2}, ε_{N_2} and b_i are in eV and m_i is in eV/O atom.

Family	parameters	value for PBE
Diatomic	ε_{O_2}	-0.46
Molecules	ε_{N_2}	0.34
N_2O_x	m_i	-0.42
	b_i	0.00
HNO_x	m_i	-0.45
	b_i	0.39
NO_x	m_i	-0.67
	b_i	0.59

Table S8 summarizes the residual DFT errors after applying the correction schemes of each study to the respective molecules using PBE. According to Table S8, all the correction schemes yield MAEs that approach chemical accuracy (~1 kcal/mol). Furthermore, the oxygen-counting procedure[4] renders comparable or lower MAE and MAX values than the sequential method and AO1. The higher MAX of the oxygen-based method against AO2 probably stems from n_o-based corrections being unable to detect the concrete chemical structures introducing the deviations.

Table S8. Residual errors obtained after applying the correction schemes in this study and in an earlier work.[4] Columns 2 to 4: the three approaches proposed here: the sequential approach (*seq*), the optimization based on chemical bonds (AO1), and the optimization that capitalizes on the errors found by the sequential approach (AO2). The last column refers to a previous correction scheme.[4] NA: not analyzed.

	this work			earlier work
species	*seq*	*AO1*	*AO2*	n_o
NH_2OH	0.00	0.09	-0.05	**NA**
NO	-0.07	0.21	-0.07	0.02
HNO	-0.04	0.24	-0.04	0.01
NO_2	0.00	-0.09	0.08	-0.04
NO_3	-0.19	-0.26	-0.07	0.02
trans-HNO_2	0.02	0.00	0.00	-0.02
cis-HNO_2	0.02	0.00	0.00	**NA**
HNO_3	0.00	0.00	0.02	0.01
N_2O	0.00	0.06	-0.07	-0.09
cis-N_2O_2	-0.10	0.00	-0.06	0.12
N_2O_3	0.06	0.04	0.07	0.06
N_2O_4	0.02	-0.14	0.00	-0.13

N_2O_5	-0.11	0.26	-0.07	0.04
MAE	0.07	0.11	0.05	0.05
MAX	0.19	0.26	0.08	0.13

S6. Enthalpic effects

A potential source of discrepancies between the experimental and DFT-calculated enthalpies of formation might be the difference in temperature, as experimental measurements are performed at 298.15 K and DFT calculations are computed at 0 K. In case these contributions are included, Equation 3 in the main text would be modified by adding the change with temperature of the formation enthalpy:

$$\Delta_f G^{DFT} \approx \Delta_f E^{DFT} + \Delta_f ZPE + (\Delta_f H_{@298K} - \Delta_f H_{@0K}) - T\Delta_f S \quad \text{(S4)}$$

For the studied dataset, the enthalpy contribution due to the change in temperature corresponds on average to -0.01 eV/atom, as shown in Table S9. The changes for N_2, H_2 and O_2 are zero at all temperatures.

Table S9. Enthalpic contributions from temperature change from 0 to 298.15 K. Numbers from refs.[6,7]

species	$\Delta_f H_{@298K} - \Delta_f H_{@0K}$ / eV	$\frac{1}{n}(\Delta_f H_{@298K} - \Delta_f H_{@0K})$ / eV atom^{-1}
NH_3	-0.07	-0.02
NH_2OH	-0.11	-0.02
NO	0.01	0.00
HNO	-0.03	-0.01
NO_2	-0.03	-0.01
NO_3	-0.05	-0.01
trans-HNO_2	-0.06	-0.02
cis-HNO_2	-0.07	-0.02
HNO_3	-0.10	-0.02
N_2O	-0.04	-0.01
cis-N_2O_2	-0.02	0.00
N_2O_3	-0.05	-0.01
N_2O_4	-0.10	-0.02
N_2O_5	-0.10	-0.01

Most enthalpic temperature corrections are smaller than -0.10 eV and there is a nearly perfect cancellation of these contributions when considering chemical reactions,

such that the change in the Gibbs energies due to temperature changes is negligible. For instance, consider the reaction: $NO_2 + NO_3 \rightarrow N_2O_5$. The change of enthalpy of this reaction from 0 to 298.15 K amounts to $-0.10 - (-0.03) - (-0.05) = -0.02\ eV$. While in general we expect errors within ±0.05 eV for the molecules in Table S9, the largest expectable error is 0.11 eV, which is the span of the values in Table S9 (i.e., NO and NH_2OH).

In conclusion, our method is based on free energies of formation, but its corrections are to be used for obtaining accurate reaction energies. Discarding enthalpic contributions due to the changes in temperature would typically lead to errors of magnitudes comparable to the MAEs and MAXs of the methods discussed in the main text. Thus, incorporating enthalpic corrections seems facultative at this point.

S7. Analysis of the initial trends in Figure 5

Equation 6 in the main text is:

$$\Delta_f G_{H_xN_yO_z}^{corrected} = \Delta_f G_{H_xN_yO_z}^{DFT} + \frac{y}{2}\varepsilon_{N_2} + \frac{z}{2}\varepsilon_{O_2} - \varepsilon_{H_xN_yO_z} \quad \text{(S5)}$$

Before the errors in the compounds are detected, $\varepsilon_{H_xN_yO_z} = 0$. Besides, for PBE $\varepsilon_{N_2} = 0.34\ eV$ and $\varepsilon_{O_2} = -0.46\ eV$. The compounds with the smallest and largest number of atoms are NO and N_2O_5, respectively. For those, Equation S5 turns into:

$$\Delta_f G_{NO}^{corrected} = \Delta_f G_{NO}^{DFT} + \frac{1 \cdot 0.34}{2} + \frac{1 \cdot -0.46}{2} = \Delta_f G_{NO}^{DFT} - 0.06\ eV \quad \text{(S6)}$$

$$\Delta_f G_{N_2O_5}^{corrected} = \Delta_f G_{N_2O_5}^{DFT} + \frac{2 \cdot 0.34}{2} + \frac{5 \cdot -0.46}{2} = \Delta_f G_{N_2O_5}^{DFT} - 0.81\ eV \quad \text{(S7)}$$

Because the offsets are in the range of -0.06 to -0.81 eV, it is likely that upon correcting O_2 and N_2 the compounds will be below the parity line in Figure 5.

S8. Direct coordinates

```
NH2OH
   1.00000000000000
    15.0000000000000000    0.0000000000000000    0.0000000000000000
     0.0000000000000000   15.0000000000000000    0.0000000000000000
     0.0000000000000000    0.0000000000000000   15.0000000000000000
   N    O    H
     1    1    3
Selective dynamics
Direct
  0.2545210566049662  0.2319941515865892  0.1360305423666130   T   T   T
  0.2214593323945584  0.2166596506120359  0.2261498078963037   T   T   T
  0.3163211844490530  0.2025649017113142  0.1366655811667420   T   T   T
  0.2667283034021186  0.2993470199524365  0.1348040884871437   T   T   T
  0.1641701231493093  0.1883676094709510  0.2146833134165339   T   T   T

NO
   1.00000000000000
    15.0000000000000000    0.0000000000000000    0.0000000000000000
     0.0000000000000000   15.0999999999999996    0.0000000000000000
     0.0000000000000000    0.0000000000000000   15.1999999999999993
   N    O
     1    1
```

```
Selective dynamics
Direct
  0.3524151333333307  0.3500812582781450  0.5570813518354012   T   T   T
  0.3524151333333307  0.3500812582781450  0.6340618718488062   T   T   T

NHO molecule
   1.00000000000000
    15.0000000000000000    0.0000000000000000    0.0000000000000000
     0.0000000000000000   15.0000000000000000    0.0000000000000000
     0.0000000000000000    0.0000000000000000   15.0000000000000000
   N    O    H
     1     1     1
Selective dynamics
Direct
  0.3544134085388595  0.3515863432024410  0.5641090392737389   T   T   T
  0.3505859503397729  0.3531724178086279  0.6451971577908531   T   T   T
  0.4184975744546958  0.3251381723222572  0.5447189362687367   T   T   T

NO2
   1.00000000000000
    15.0000000000000000    0.0000000000000000    0.0000000000000000
     0.0000000000000000   15.0000000000000000    0.0000000000000000
     0.0000000000000000    0.0000000000000000   15.0000000000000000
   N    O
     1     2
Selective dynamics
Direct
  0.2665630819362343  0.2665728039580101  0.2664213476620861   T   T   T
  0.2665864937767498  0.2665937667348960  0.3472748499654109   T   T   T
  0.3089837576203453  0.3066334293070978  0.2104371357058316   T   T   T

NO3
   1.00000000000000
    15.0000000000000000    0.0000000000000000    0.0000000000000000
     0.0000000000000000   15.0000000000000000    0.0000000000000000
     0.0000000000000000    0.0000000000000000   15.0000000000000000
   N    O
     1     3
Selective dynamics
Direct
  0.3350143456256398  0.3333333333333357  0.3216687587598528   T   T   T
  0.3355448717761098  0.3333333333333357  0.4050962000790669   T   T   T
  0.2624615202401691  0.3333333333333357  0.2804536443560531   T   T   T
  0.4069792623580847  0.3333333333333357  0.2794480634717031   T   T   T

trans-HNO2
   1.00000000000000
    15.0000000000000000    0.0000000000000000    0.0000000000000000
     0.0000000000000000   15.0000000000000000    0.0000000000000000
     0.0000000000000000    0.0000000000000000   15.0000000000000000
   H    N    O
     1     1     2
Selective dynamics
Direct
  0.4550728482713578  0.3333333333333357  0.3145300225092535   T   T   T
  0.3274388736511047  0.3333333333333357  0.3241667716761455   T   T   T
  0.2626697607828568  0.3333333333333357  0.2790211394291751   T   T   T
  0.4081518506280209  0.3333333333333357  0.2689487330520934   T   T   T

cis-HNO2
   1.00000000000000
    15.0000000000000000    0.0000000000000000    0.0000000000000000
     0.0000000000000000   15.0000000000000000    0.0000000000000000
     0.0000000000000000    0.0000000000000000   15.0000000000000000
   H    N    O
     1     1     2
Selective dynamics
Direct
  0.3871415633090916  0.3206150622134859  0.2078595075639293   T   T   T
  0.3290579706932107  0.3368805488786859  0.3219732105790901   T   T   T
  0.2618070285019451  0.3310010053280929  0.2792770663956440   T   T   T
  0.4078601041624175  0.3292367169130785  0.2700902154613439   T   T   T

HNO3
   1.00000000000000
    15.0000000000000000    0.0000000000000000    0.0000000000000000
     0.0000000000000000   15.0000000000000000    0.0000000000000000
     0.0000000000000000    0.0000000000000000   15.0000000000000000
   H    N    O
     1     1     3
Selective dynamics
Direct
  0.4540361216681847  0.3333333333333357  0.3154283751440832   T   T   T
  0.3272540137712939  0.3333333333333357  0.3253168055757527   T   T   T
  0.3404467358673398  0.3333333333333357  0.4058418142976744   T   T   T
  0.2585840479307271  0.3333333333333357  0.2829960622771717   T   T   T
  0.4063457474291233  0.3333333333333357  0.2704169427053193   T   T   T

N2O
   1.00000000000000
    15.0000000000000000    0.0000000000000000    0.0000000000000000
     0.0000000000000000   15.0000000000000000    0.0000000000000000
     0.0000000000000000    0.0000000000000000   15.0000000000000000
   N    O
     2     1
Selective dynamics
Direct
  0.1333333333333329  0.1333333333333329  0.1313961184243681   T   T   T
  0.1333333333333329  0.1333333333333329  0.2076798747228810   T   T   T
  0.1333333333333329  0.1333333333333329  0.2875906735194160   T   T   T

N2O2
   1.00000000000000
    15.0000000000000000    0.0000000000000000    0.0000000000000000
     0.0000000000000000   15.0000000000000000    0.0000000000000000
     0.0000000000000000    0.0000000000000000   15.0000000000000000
   N    O
     2     2
Selective dynamics
Direct
  0.1337409318356768  0.1309068685053408  0.1147823520886360   T   T   T
  0.1291848173588392  0.1303579729888522  0.2469295565260433   T   T   T
  0.1719472329209596  0.1885459442908094  0.2770703096789761   T   T   T
  0.1782603512178547  0.1894558808816618  0.0880844483730107   T   T   T

N2O3
   1.00000000000000
    15.0000000000000000    0.0000000000000000    0.0000000000000000
     0.0000000000000000   15.0000000000000000    0.0000000000000000
     0.0000000000000000    0.0000000000000000   15.0000000000000000
   N    O
     2     3
Selective dynamics
Direct
  0.1008629370090849  0.1353244618892805  0.2471591212874925   T   T   T
  0.0886926296808923  0.1287903183234719  0.3750459599323455   T   T   T
  0.1324206766519977  0.1865222975248595  0.4118369249787899   T   T   T
  0.0408900659931107  0.0705391617984723  0.4051608465568152   T   T   T
  0.1481156576061446  0.1949290064800999  0.2339710978044920   T   T   T

N2O4
   1.00000000000000
    15.0000000000000000    0.0000000000000000    0.0000000000000000
     0.0000000000000000   15.0000000000000000    0.0000000000000000
     0.0000000000000000    0.0000000000000000   15.0000000000000000
   N    O
     2     4
Selective dynamics
Direct
  0.1006598686751027  0.1347552351336739  0.2526987894799427   T   T   T
  0.0893311612558376  0.1296462885044061  0.3766963598770224   T   T   T
  0.1320285216857300  0.1865135299824210  0.4142065540559327   T   T   T
  0.0409739665898779  0.0701839253930850  0.4009577920680645   T   T   T
  0.1480265985259573  0.1949537051611804  0.2282896830353854   T   T   T
  0.0589132166008258  0.0770806491585706  0.2153508214836428   T   T   T

N2O5
   1.00000000000000
    15.0000000000000000    0.0000000000000000    0.0000000000000000
     0.0000000000000000   15.0000000000000000    0.0000000000000000
     0.0000000000000000    0.0000000000000000   15.0000000000000000
   N    O
     2     5
Selective dynamics
Direct
  0.1725076651526237  0.3182877312999081  0.3384650418997312   T   T   T
  0.3517191810176917  0.3336254471431025  0.3323111704961911   T   T   T
  0.4086641263052806  0.3358959662248587  0.2756950475220403   T   T   T
  0.3529487404353254  0.3360806257555481  0.4123657784676235   T   T   T
  0.2603227670003767  0.3268213687556010  0.2838161707124082   T   T   T
  0.1769240775334450  0.3137198611848321  0.4183101804918463   T   T   T
  0.1117801092219271  0.3172356663028160  0.2859032770768299   T   T   T
```

References

(1) Haynes, W. M.; Lide, D. R.; Bruno, T. J. *CRC Handbook of Chemistry and Physics*, 97th ed.; CRC Press/Taylor And Francis: Boca Raton, FL, 2016. https://doi.org/10.1201/9781315380476.

(2) Linstrom, P. J.; Mallard, W. G. *NIST Chemistry WebBook, NIST Standard Reference Database Number 69*; National Institute of Standards and Technology: Gaithersburg MD, 20899, 2022. https://doi.org/10.18434/T4D303.

(3) GAMS Development Corporation. *GAMS Documentation 39.1.1*. https://www.gams.com/latest/docs/ (accessed 2022-06-04).
(4) Urrego-Ortiz, R.; Builes, S.; Calle-Vallejo, F. Impact of Intrinsic Density Functional Theory Errors on the Predictive Power of Nitrogen Cycle Electrocatalysis Models. *ACS Catal.* **2022**, *12* (8), 4784–4791. https://doi.org/10.1021/acscatal.1c05333.
(5) Kurth, S.; Perdew, J. P.; Blaha, P. Molecular and Solid-State Tests of Density Functional Approximations: LSD, GGAs, and Meta-GGAs. *Int. J. Quantum Chem.* **1999**, *75* (4-5), 889–909. https://doi.org/10.1002/(SICI)1097-461X(1999)75:4/5<889::AID-QUA54>3.0.CO;2-8.
(6) Ruscic, B.; Bross, D. H. *Active Thermochemical Tables (ATcT) values based on ver. 1.122 of the Thermochemical Network (2016)*. https://atct.anl.gov/ (accessed 2022-08-05).
(7) Ruscic, B.; Pinzon, R. E.; Laszewski, G. von; Kodeboyina, D.; Burcat, A.; Leahy, D.; Montoy, D.; Wagner, A. F. Active Thermochemical Tables: Thermochemistry for the 21st Century. *J. Phys. Conf. Ser.* **2005**, *16*, 561–570. https://doi.org/10.1088/1742-6596/16/1/078.
